# Trends in Prevalence of Opioid Dependence in Scotland 2014–2023: Multi‐Parameter Estimation of Prevalence

**DOI:** 10.1111/dar.70256

**Published:** 2026-09-14

**Authors:** Andreas Markoulidakis, Matthew Hickman, Megan Glancy, Andrew McAuley, Lee R. Barnsdale, Tara Shivaji, Craig Collins, Jaroslaw Lang, Femke de Wit, Gordon Hunt, Sharon J. Hutchinson, Louisa Degenhardt, Hayley E. Jones

**Affiliations:** ^1^ Bristol Medical School University of Bristol Bristol UK; ^2^ Public Health Scotland Edinburgh UK; ^3^ Glasgow Caledonian University Glasgow UK; ^4^ National Drug and Alcohol Research Centre UNSW Sydney Sydney Australia; ^5^ Clinical and Protecting Health Directorate Public Health Scotland Edinburgh UK; ^6^ Health Harms Intelligence Team Public Health Scotland Edinburgh UK; ^7^ National Drug & Alcohol Research Centre Faculty of Medicine and Health, UNSW Sydney Sydney Australia; ^8^ Population Health Sciences Bristol Medical School, University of Bristol Bristol UK

**Keywords:** Bayesian methods, indirect estimation, multi‐parameter estimation of prevalence, opioid agonist therapy, people with opioid dependence

## Abstract

**Introduction:**

Scotland remains in the midst of a public health crisis driven by drug‐related deaths, with rates reaching 2.0 per 100 person‐years in 2020–22. Estimates of the prevalence of opioid dependence are critical for supporting policy decisions and better understanding the factors behind this rise.

**Methods:**

We applied a Bayesian multi‐parameter estimation of prevalence approach to estimate the prevalence of opioid dependence from 2014/15 to 2023/24. We used linked opioid agonist therapy prescriptions, mortality and hospital admission data from the Substance Use and Health Intelligence Linked Dataset.

**Results:**

We estimate there were 43,600 (95% credible intervals 42,000–45,400) people with opioid dependence in Scotland in 2023/24, which is 1.13% (95% credible intervals 1.09%–1.18%) of those aged 15–69. Opioid agonist therapy coverage was estimated as 61%. Estimated prevalence was higher among those aged 50–69 (0.76%) than 15–34 (0.67%). Prevalence varied across regions, being highest in Greater Glasgow & Clyde (1.46%). Over the 10‐year period, the estimated population size decreased by 4100 (95% credible intervals 6800 –1500).

**Discussion and Conclusions:**

In Scotland, prevalence of opioid dependence remains high. There are signs of a modest decline but, notably, the reduction is less than the number of opioid‐related poisoning deaths over the period modelled.

## 1 Introduction

Scotland continues to face a major public health emergency related to drug‐related deaths (DRD; i.e., deaths involving use of illegal drugs or misuse of legal drugs), with the emergency formally recognised by the Scottish Government in 2021 [1, 2]. In the decade preceding the COVID‐19 pandemic, the number of DRDs more than tripled from 368 in 2011–12 to 1318 in 2019–20 [3]. Although DRDs stabilised during the pandemic period and dropped 23% between 2021 and 2024 [4], they remain among the highest in Europe, with rates of 19.8 DRD per 100,000 population, approaching those observed in North America [5]. DRD rates among people dependent on opioids have nearly trebled, from 0.7 per 100 person years in 2011–13 to 2.0 per 100 person years in 2020–22 [6]. Although the risk of DRD increases with age, earlier work suggests that population ageing alone does not fully explain the observed increase in mortality [7].

The factors underlying the rise in DRDs and mortality risk among people with opioid dependence are complex and multifactorial [8]. Increased numbers of DRDs or rates in the general population alone are difficult to interpret, as they do not distinguish between an increasing mortality risk among a stable population, reduced treatment coverage, or genuine changes in the size of the population of people with opioid dependence. Reliable estimates of population size are therefore critical for understanding the drivers of the DRD epidemic. In Scotland, previous analyses suggest that the overall population of people with opioid dependence did not increase during the period of rising DRD numbers, pointing instead to increasing mortality risk as the primary driver [3, 7]. Opioid agonist therapy (OAT) is strongly protective—with an estimated 70% reduction in DRD risk during periods on compared to periods off OAT on average—but in Scotland, absolute DRD risk has increased over time for people both on and off OAT, even as this relative protective effect of OAT has been maintained [3, 9]. This suggests that factors beyond treatment coverage alone were driving the rise in mortality risk in this population. A strong association has been observed between increasing DRDs and higher levels of polydrug use, particularly the growing presence of non‐prescribed (‘street’) benzodiazepines detected at post‐mortem [10].

In this paper, we use the Multi‐Parameter Estimation of Prevalence (MPEP) approach [11, 12] to generate estimates of opioid dependence prevalence over a 10‐year period (2014/15 to 2023/24), for Scotland overall and for nine Scottish health boards. This updates and extends our previous work, in which we estimated prevalence in three health boards (and Scotland overall) over the period 2014/15 to 2019/20 [13]. Extending the time series to 2023/24 allows us to examine recent trends in prevalence of opioid dependence, assessing whether the recent reduction in DRDs reflects a change in the population size, and providing policymakers and service planners with contemporary estimates. Improvements to the model enabled us to increase the geographical granularity of this prevalence estimation, while improvements to the underlying data linkage have enabled more accurate classification of treatment episodes.

## 2 Methods

### 2.1 Overview of MPEP

MPEP is an indirect estimation technique that utilises health and other administrative data. It is conceptually related to other indirect methods, such as simple multiplier methods or capture‐recapture, which infer prevalence from assumptions about the proportion of the population identified by a particular data source or the overlap of individuals across multiple data sources [14]. However, MPEP takes an all available evidence approach, requiring event rate estimates that are specific to different population sub‐groups, with more thorough testing of evidence inconsistency and violation of underlying assumptions.

This is implemented through a Bayesian modelling framework [11, 13, 15], built on established principles of evidence synthesis [16, 17]. MPEP begins with identification of a large ‘baseline cohort’ of individuals known (through treatment records) to be dependent on opioids, with administrative records linked to event data. The number of additional people dependent on opioids—and hence the total population size or prevalence—is estimated from numbers of specific events occurring outside of this cohort, via assumptions about the relationships between event rates in vs. outside of the cohort [11, 12, 13, 15].

Model assumptions include: (i) the events modelled are highly specific to people with opioid dependence, such that all events can be assumed to occur within this population; (ii) the baseline cohort includes all individuals receiving OAT; (iii) all members of the cohort remain opioid‐dependent throughout their follow‐up period until censoring; and (iv) within each demographic group and year, event rates among the population outside the baseline cohort are equal to the rates observed among the baseline cohort during periods off OAT [11, 13, 15, 18]. Note that the first assumption requires restricting analyses to event types that can reasonably be assumed to occur only among people with opioid dependence. Broad categories, such as all drug‐related deaths, are not appropriate for MPEP modelling because they lack sufficient specificity. For a full description of the MPEP approach see Markoulidakis et al. [12].

We applied MPEP to estimate prevalence of opioid dependence stratified by age group (15–34, 35–49, 50–69 years), sex (females, males), financial year (2014/15 to 2023/24), and nine regions (NHS Boards). The remaining five NHS Boards (NHS Borders, Highland, Orkney, Shetland and Western Isles), which cover 9% of the population aged 15–69, were consolidated as “Rest of Scotland” due to low event counts.

### 2.2 Data

We used data from the Substance Use and Health Intelligence Linked Dataset [19], which includes (among other data) administrative records on all individuals receiving OAT prescriptions in the community in Scotland, opioid‐related mortality and hospital admissions data. For application of MPEP, we defined the baseline cohort for each financial year as comprising individuals aged 15–69 years living in Scotland who received at least one OAT prescription in the current or previous 4 years. Follow‐up began on 1 April 2014 for those receiving OAT at that date or who had received OAT during the 4 years prior, or at first subsequent OAT prescription otherwise. Follow‐up was censored at the earliest of death, migration out of Scotland, 4 years after last treatment, or 31 March 2024. This cohort definition assumes opioid dependence persists for at least 4 years after last treatment. All observation time was classified as ‘on’ or ‘off’ OAT using an established algorithm [13, 20]. Two types of events were modelled: (i) opioid‐related deaths; and (ii) opioid‐related hospital admissions. We did not include all overdose deaths but only those with ICD‐10 codes indicating dependence and toxicological evidence that heroin/morphine, methadone or buprenorphine were implicated. Similarly, we included only hospital admissions with ICD‐10 codes indicating poisoning by opium, heroin or methadone. Full definitions are provided in Appendix A. Events and person‐time at risk were aggregated by sex, age group, year, treatment status and region for modelling.

### 2.3 Statistical Analysis

Opioid‐related deaths and hospital admissions within the baseline cohort were modelled using Poisson regressions with follow‐up time as an offset. These regressions included main effects for year (as indicator variables), sex, age group, treatment status, and region, as well as an interaction between treatment status and year, motivated by prior evidence of treatment–year effects on drug‐related deaths [3]. Additional interactions were included where supported by model fit criteria. Counts of events occurring outside the baseline cohort were also assumed to follow Poisson distributions. Event rates among unobserved individuals with opioid dependence were assumed to equal those observed in the cohort during periods off OAT, within strata defined by sex, age, year, and region. The unobserved person‐time at risk was modelled as a function of the ‘extra’ prevalence (this is, the number of opioid dependent people who were not observed in the baseline cohort, as a percentage of the general population), which followed a regression structure on the log‐odds scale with main effects for sex, age group, year, and region and additional interactions guided by model fit. Prevalence was estimated jointly using opioid‐related deaths and hospital admissions, facilitating consistency checks [12, 13, 15].

All models were fitted within a Bayesian framework using Stan [21], implemented via R, with model selection informed by the Deviance Information Criterion, where lower values indicate better fit after penalising for model complexity. A Bayesian approach allows the simultaneous estimation of multiple linked regression models, including latent prevalence. Vague prior distributions were specified for all parameters.

### 2.4 Sensitivity Analysis

Because prevalence estimates were based on joint modelling of opioid‐related deaths and hospital admissions, we conducted sensitivity analyses by excluding each data source in turn. An additional sensitivity analysis shortened follow‐up in the baseline cohort, censoring individuals 1–2 years after their last OAT prescription rather than 4–5 years.

## 3 Results

**FIGURE 1:**
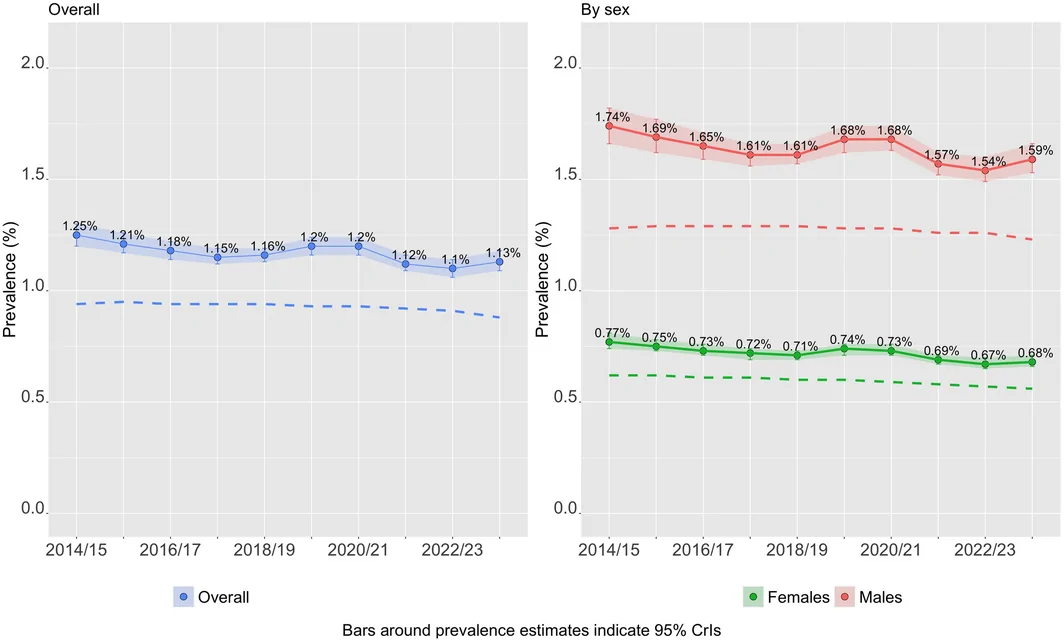
Estimated prevalence of opioid dependence among the population aged 15–69 years in Scotland; overall and by sex; 2014/15 to 2023/24. The dashed lines represent the size of the baseline cohort as a percentage of the general population. CrI, credible interval.

Figure 1 and Table B1 (Appendix B) show the estimated prevalence of opioid dependence in the Scottish population aged 15–69 years (overall and by sex) from 2014/2015 to 2023/2024. Further stratification (by age, sex, year and NHS Board) is available online at Public Health Scotland.

The overall prevalence and number of people with opioid dependence aged 15–69 years in 2023/24 were estimated as 1.13% (95% credible intervals [CrI] 1.09%–1.18%) and 43,600 (95% CrI 42,000–45,400), respectively. Prevalence was estimated at 1.59% (95% CrI 1.53%–1.66%) in men and 0.68% (95% CrI 0.66%–0.71%) in women during that year. In each of the 10 years, around two‐thirds of people with opioid dependence in Scotland were male.

Coverage or annual exposure to OAT was high—with an estimated 65% of people with opioid dependence in 2023/24 having received at least one OAT prescription during that year and a further 13% having received OAT during the four preceding years. OAT exposure was estimated to be slightly higher in females than males (Table B1, Appendix B).

Overall, there was evidence of a small reduction in overall prevalence of 4100 (95% CrI 6800–1500) since 2014/15. Over the same time period there were 5834 opioid‐related poisoning deaths in Scotland, two‐thirds (3889) of which were observed within the baseline cohort. Note that these are numbers of deaths restricted to the specific definitions used in this study: total numbers of opioid‐related deaths are higher. There were also an additional 5933 deaths from other causes in the cohort over the 10‐year period, therefore likely a total of over 14,000 deaths among people with opioid dependence over this time period.

**FIGURE 2:**
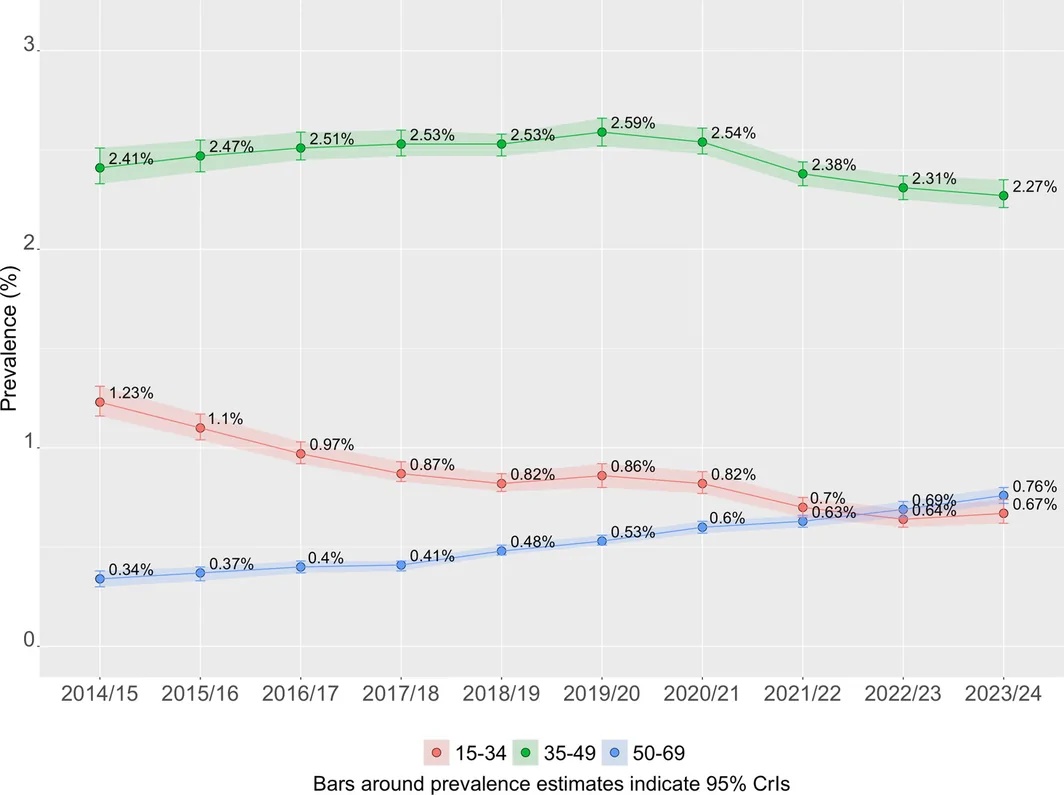
Estimated prevalence of opioid dependence among the population aged 15–69 years in Scotland; by age‐group; 2014/15 to 2023/24. CrI, credible interval.

Figure 2 shows estimated prevalence by age group. Across the 10‐year period, the highest estimated prevalence was observed among men aged 35–49 years. While prevalence among those aged 15–34 years was higher than among those aged 50–69 years in earlier years, this pattern reversed from 2022/23 onwards, with higher estimated prevalence in the older age group.

**FIGURE 3:**
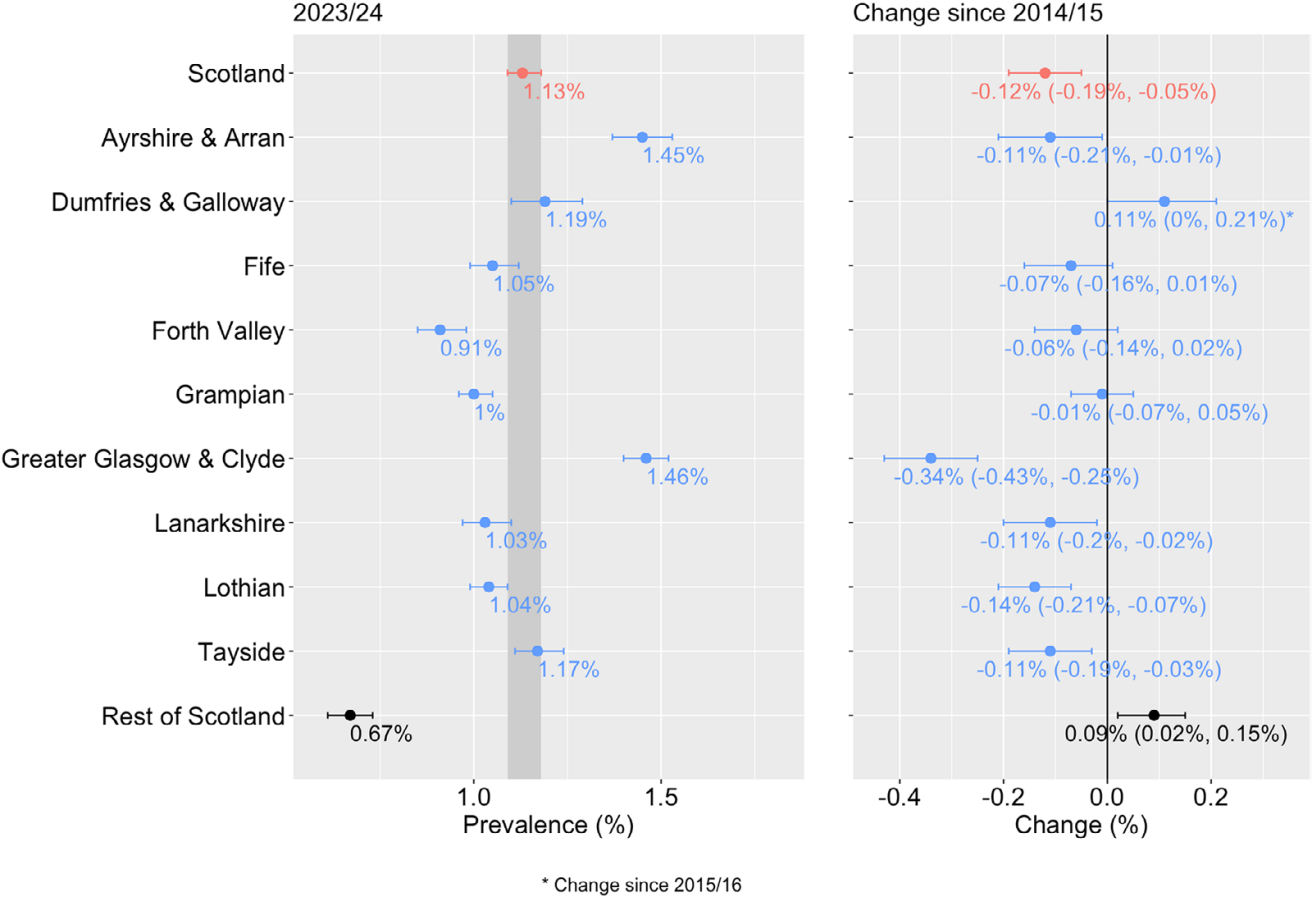
Estimated prevalence of opioid dependence among the population aged 15–69 years in Scotland; by NHS Board; 2023/24. The plot also shows the estimated absolute change in prevalence between 2014/15 and 2023/24 for each NHS Board. *For NHS Dumfries & Galloway we report estimated change since 2015/16, due to concerns about the reliability of the 2014/15. OAT prescription data in this region (a high proportion of prescriptions were missing identifiers).

**FIGURE B1:**
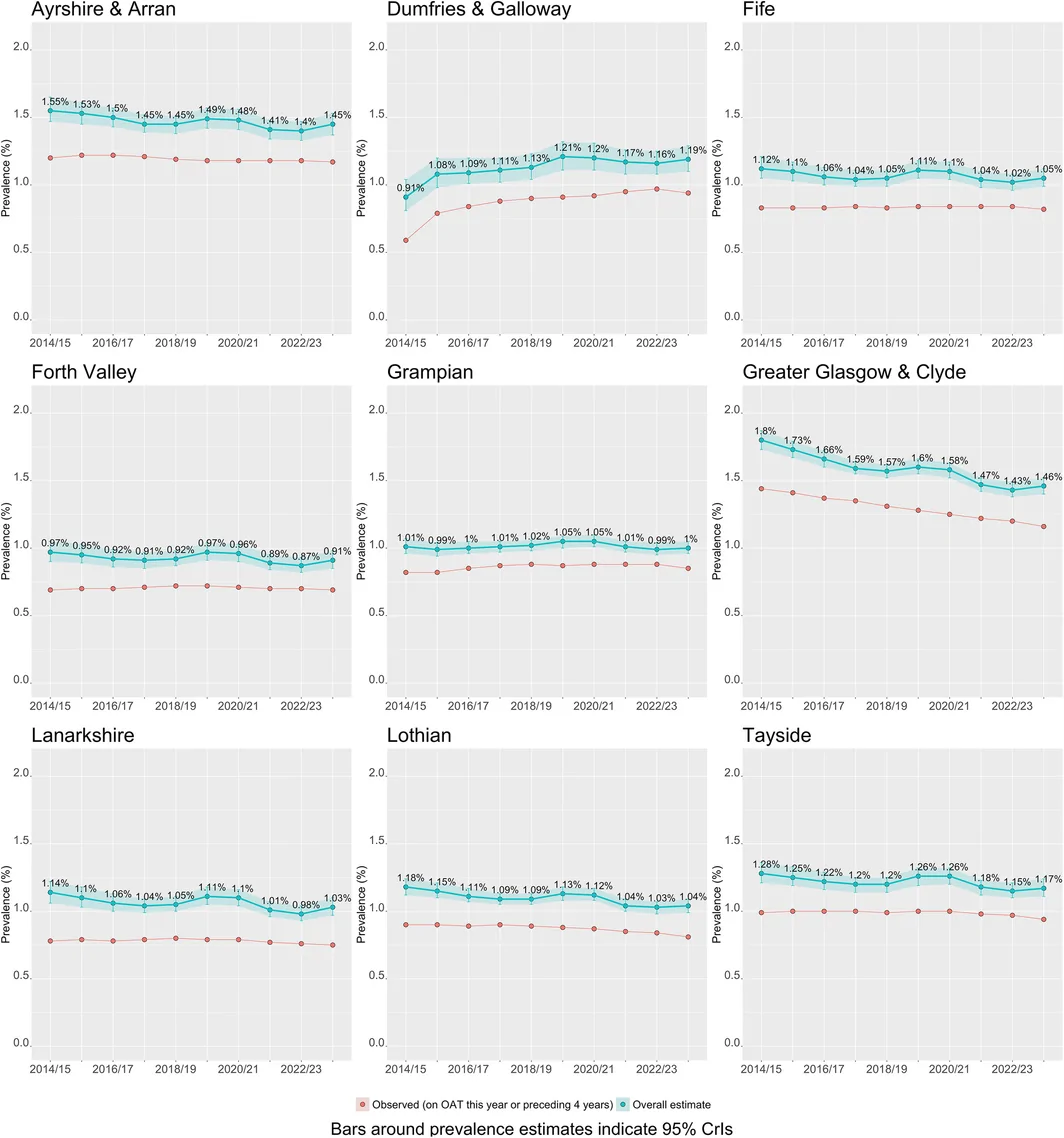
Estimated prevalence of opioid dependence among the population aged 15–69 years in Scotland; by region; 2014/15 to 2023/24. CrI, credible interval.

MPEP generated estimates for nine of the 14 health boards in Scotland, areas that covered 91% of the general population (Figure 3). The regions ranged in size from 148,000 people in NHS Dumfries and Galloway to 1.2 million in Greater Glasgow & Clyde. The estimated prevalence of opioid dependence varied from 0.91% (95% CrI 0.85%–0.98%) in NHS Forth Valley to 1.46% (95% CrI 1.40%–1.52%) in NHS Greater Glasgow & Clyde in 2023/2024. The figure also shows the estimated absolute percentage change in each region between 2014/2015 and 2023/2024. Most of the estimated reduction in population size over the 10‐year period was in NHS Greater Glasgow & Clyde: the number of people with opioid dependence was estimated to have reduced by 2300 (95% CrI 1500–3000), an absolute reduction in prevalence of 0.34% (95% CrI 0.25%–0.45% reduction) between 2014/15 and 2023/24. Estimated prevalence over time for each region is depicted in Figure B1, Appendix B: we see some evidence of smaller reductions in some other regions.

### 3.1 Sensitivity Analysis

**FIGURE 4:**
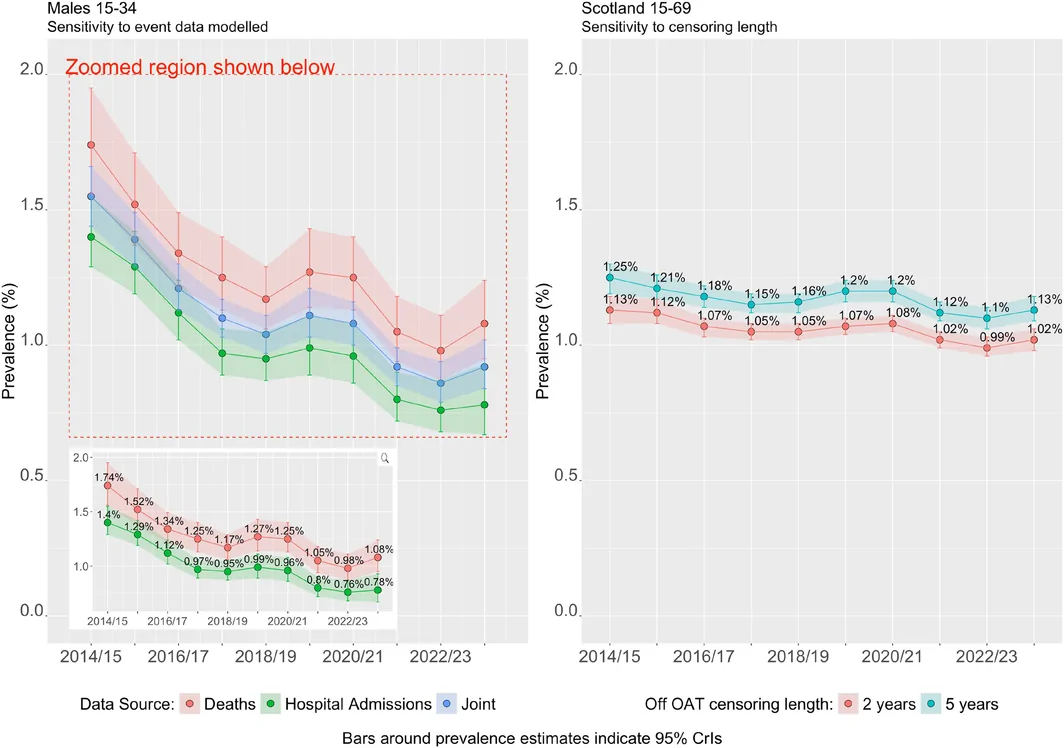
Results from sensitivity analyses. Left panel: Analyses based on modelling the two event types separately. Estimated prevalence of opioid dependence among the male population aged 15–34 years in Scotland; by age‐group; 2014/15 to 2023/24. The zoomed plot depicts only the estimates from single event types, to highlight the discrepancies. Right: Estimated prevalence of opioid dependence; Comparison of results with censoring at 4–5 years since last OAT prescription (primary analysis) or 1–2 years (sensitivity analysis); 2014/15 to 2023/24; CrI, credible interval; OAT, opioid agonist therapy.

In sensitivity analyses based on modelling of each event type separately, overall prevalence estimates did not differ substantially from the primary analysis (see Appendix B, Table B2). However, slight differences emerged for young males, for whom 95% CrIs around estimated prevalence based on individual event types did not overlap for some years (Figure 4; left). For this group, prevalence estimates based on hospital admissions alone were consistently lower than those derived from mortality data alone.

Prevalence estimates were sensitive to choice of censoring rule. Figure 4 (right) shows prevalence estimates from the sensitivity analysis in which follow‐up was censored at the end of the financial year lying between one and 2 years since last OAT. Results are plotted alongside those from the primary analysis. The absolute difference in estimates across these two analyses is between 0.09% and 0.12% for each year, which corresponds to between 4000 and 5000 people. This discrepancy is driven by differences in event rates among individuals ‘off OAT’ across these two analyses, as the shorter censoring period results in higher estimated rates.

## 4 Discussion

### 4.1 Main Finding of This Study

We estimate that in 2023/24 there were 43,600 (95% CrI 42,000–45,400) people with opioid dependence in Scotland—a prevalence of 1.13% (95% CrI 1.09%–1.18%) of those aged 15–69 years, with annual OAT coverage estimated to be 59%–64%. There was an estimated 0.16% (95% CrI 0.09%–0.22%) reduction in absolute prevalence over a 10‐year period: a reduction of 4100 people with opioid dependence. The population of people with opioid dependence is ageing and continues to be exposed to considerable mortality risk. The estimated 4100 reduction in population size should be interpreted alongside there having been over 5000 opioid‐related poisoning deaths and likely more than 14,000 all cause deaths among people with opioid dependence over the same 10‐year period.

In 2022/23, for the first time, the estimated number of people with opioid dependence aged over 50 years (10,200; 95% CrI 9700–10,800) exceeded the number aged 15–34 years (8600; 95% CrI 8000–9300), a clear signal of an ageing epidemic of opioid use. Furthermore, this suggests that onset of opioid dependence among younger people is occurring at a slower pace than outflow through long‐term remission/cessation or death—with important implications for services needing to care for an ageing population that may increasingly be experiencing a range of comorbid physical health problems.

To help illustrate the intuition behind the MPEP estimate, we can apply a crude multiplier method to estimate the number of people who were opioid dependent and not captured in the cohort in 2023/24, and add this to the size of the baseline cohort (~34,000). There were 176 opioid‐related poisoning deaths in the non‐cohort, while 201 were observed in the cohort during 10,800 “off treatment” person years at risk. A crude estimate of population size is then 34,000 + 176/(201/10,800)≈43,500—which is close to the MPEP estimate. Note that, although this crude estimate is a multiplier, it is atypical in that it is not a naïve one: linked data have been used to derive a benchmark and mortality rate that are carefully matched by location, year and treatment status. The MPEP further substantially improves upon this by joint modelling of deaths and hospitalisations, allowing for uncertainty in both the observed event counts and event rates, accommodating heterogeneity through covariates, facilitating assessment of consistency of evidence across data sources, and providing a flexible framework for incorporating additional evidence where violations of assumptions are identified.

### 4.2 What Is Already Known on This Topic

There are few regularly updated robust national estimates of the prevalence of opioid dependence in other countries. In Europe it was estimated that prevalence was 0.3% in 2022, but this was based on multiple estimates generated using different methods, each subject to its own biases, and over half of the countries were missing any or recent estimates after 2020 [22, 23]. Similarly, global estimates of the prevalence of people who inject drugs (PWID: 14.8 million people aged 15–64 in 2021) or opioid dependence (more than 36 million people aged 15–64 in 2021) rely upon pooling across different methods and years [24, 25].

### 4.3 What This Study Adds

Our new estimates extend earlier prevalence estimates in Scotland confirming that the high number of drug‐related deaths is not due to an increase in prevalence of opioid dependence but driven by increases in mortality risk. Equally, our estimates suggest that the recent reduction in DRDs in Scotland (a 23% decrease from 2021 to 2024) [4] cannot be fully explained by a decline in the underlying prevalence of opioid dependence but also reflects a stabilisation and potential reduction in mortality risk in the population.

### 4.4 Limitations

Our estimates are based on direct observation of a large cohort and estimation of the additional number of people with opioid dependence outside of this cohort. This is jointly informed by two event types, which are in agreement on overall prevalence.

However, as with any indirect method, bias may arise if key assumptions are violated. Potential sources include incomplete capture of individuals receiving OAT, misclassification of opioid‐related events, and differences in event rates between populations in and out of the baseline cohort. Some risks have been mitigated since our initial application of MPEP to Scotland [13], through improved linkage across data sources and updates to historical prescribing records, enabling more accurate definition of treatment episodes. Nevertheless, inconsistencies between estimates derived from different event types remain among men aged 15–34 years, where hospitalisation data implied lower prevalence than mortality data. We additionally note that estimated OAT coverage was lower for this group. In contrast, estimates were broadly consistent overall, and for women and older age groups. The reasons for discrepancies in estimates for younger men require further investigation. One possibility is greater misclassification or incomplete capture of younger men in OAT records, which could arise due to Substance Use and Health Intelligence Linked Dataset currently only capturing prescriptions in the community, and missing OAT provision in prison settings. Younger men with opioid dependence have relatively high incarceration rates, such that omission of prison‐based treatment could lead to substantial misclassification of follow‐up time as ‘off’ versus ‘on’ OAT in this group. We aim to address this in future work by linking in prison data, allowing us to include OAT episodes among those in prison [9].

Furthermore, MPEP assumes modelled events occur exclusively among people with opioid dependence. In practice, however, there may be some level of misclassification. For example, people unknowingly exposed to nitazene or fentanyl‐contaminated non‐opioid drugs are an ongoing problem in North America [26] and a growing concern in Scotland [27]. Such events would be incorrectly attributed to the population of interest, biasing prevalence estimates upward. We expect this bias to be minor historically in Scotland, but additional priors and bias parameters will be needed in the future and in other settings.

It should be noted that the 95% credible intervals presented throughout reflect statistical uncertainty conditional on the model assumptions being correct. The intervals are notably narrow, partly reflecting the large volume of administrative data used. True uncertainty is likely wider, however, due to uncertainty arising from potential model misspecification.

In principle, the choice of censoring length should not affect MPEP prevalence estimates. However, sensitivity analyses indicated lower prevalence when the censoring period (time since last treatment) was reduced to 2 years. This discrepancy was due to off‐treatment event rates being estimated as higher in this analysis, such that events out of the cohort are attributed to a smaller population size—thereby reducing estimated prevalence. The difference in event rates likely reflects the concentration of events shortly after treatment cessation (particularly within the first 4 weeks)—although potential cessation of opioid dependence among some individuals could also affect this. We are working on improving the quality of prescription data to further refine the classification of treatment periods and potentially account for increased mortality during the initial period after OAT cessation.

Although empirical evidence to fully evaluate some assumptions is limited, historical data from cohort studies that include people not exposed to OAT offer partial reassurance that event rates are similar [28]. Future work will incorporate additional indicators of non‐fatal overdose as a third event type, such as emergency department presentations, to strengthen inference, allow further consistency checks, and improve robustness of the estimates. Future work will also extend the model to estimate PWID prevalence and refine age groupings.

### 4.5 Implications

Although many indirect estimation methods provide limited opportunity for external validation or explicit assessment of their assumptions, the MPEP approach leverages linked administrative data and multiple event types within the population of interest, strengthening both internal consistency and credibility. As additional information becomes available—such as treatment records from prison settings—it can be incorporated into future model iterations or used to evaluate key assumptions, enabling further refinement and mitigation of potential sources of bias.

Our prevalence estimates are critical to understanding trends in drug‐related harm. For example, these estimates are crucial to evaluations of hepatitis C (HCV) treatment scale‐up and converting public health surveillance data into projections of HCV incidence and whether Scotland is meeting World Health Organization elimination targets [29].

In Scotland, our estimates, alongside information on mortality rates, suggest that incidence is lower than the combination of cessation and mortality. The population of people dependent on opioids is ageing, with increased comorbidity and health and social care needs. For instance, over the 10 years there were 5834 opioid‐related deaths and likely over 14,000 all cause deaths in people dependent on opioids, which is substantially greater than the estimated reduction in population size over the same period.

While our model does not yet directly estimate inflow to or outflow from the population of people with opioid dependence—although it could be extended to a multi‐state model which could produce these (as has been developed in ecology) [27]—our estimates allow an approximate decomposition of population dynamics over the decade, assuming migration in and out of Scotland roughly cancels. Over the 10‐year period, the difference between total outflow and total inflow was approximately 4000. Given that the population experienced approximately 14,000 deaths from any cause, the difference between total initiations and total cessations was approximately 10,000 people—or around 1000 more initiations than cessations per year. However, more detailed incidence estimates can only come once uncertainty has been reduced over long‐term cessation rates from opioid use disorders.

Our findings have direct implications for public health policy and practice. There remains a significant intervention gap in Scotland—with slowly declining prevalence of people with opioid dependence, relatively high exposure to OAT and national naloxone distribution [30], but still a disproportionately high burden of overdose and other mortality. A supervised injecting room is being piloted in Glasgow [31] but more needs to be done to extend OAT retention and reduce mortality risk in the community. In contrast, Scotland has made substantial progress towards eliminating HCV as a public health problem—which also benefited from robust evidence on population size and good public health surveillance underpinning the scale‐up of HCV treatment in the community [32]. Patterns of drug use in Scotland and globally are changing—with increases in polydrug use and mixing of routes of consumption. For example, in Scotland there has been an increase in the proportion of fatal opioid‐related overdoses detecting benzodiazepines and/or cocaine (56% and 47% respectively in 2024) [4]; and increases observed in cocaine injecting among PWID and people on OAT [33]. Furthermore, in parts of North America, where DRD numbers have risen dramatically, it remains unclear which of these mechanisms predominates—highlighting the urgent need for robust population size estimates that contextualise. There is an urgent need to extend MPEP to other forms of problem drug use—especially substance use disorder—as well as generate estimates of prevalence of PWID.

## Author Contributions

**Andreas Markoulidakis:** data curation (equal); formal analysis (lead); methodology (equal); software (lead); visualisation (lead); writing – original draft (equal); writing – review and editing (lead). **Matthew Hickman:** conceptualisation (equal); funding acquisition (equal); methodology (equal); supervision (supporting); writing – original draft (equal); writing – review and editing (equal). **Megan Glancy:** data curation (equal); software (supporting); writing – review and editing (supporting). **Andrew McAuley:** conceptualisation (supporting); funding acquisition (equal); supervision (supporting); writing – original draft (supporting); writing – review and editing (supporting). **Lee R. Barnsdale:** conceptualisation (equal); data curation (equal); funding acquisition (equal); project administration (lead); supervision (equal); writing – review and editing (supporting). **Tara Shivaji:** conceptualisation (equal); funding acquisition (equal); project administration (equal); supervision (supporting). **Craig Collins:** project administration (equal); writing – review and editing (supporting). **Jaroslaw Lang:** data curation (equal); writing – review and editing (supporting). **Femke de Wit:** data curation (equal); writing – review and editing (supporting). **Gordon Hunt:** data curation (equal); writing – review and editing (supporting). **Sharon J. Hutchinson:** conceptualisation (equal); funding acquisition (equal); supervision (supporting); writing – review and editing (supporting). **Louisa Degenhardt:** conceptualisation (equal); funding acquisition (equal); supervision (supporting); writing – review and editing (supporting). **Hayley E. Jones:** conceptualisation (equal); funding acquisition (equal); methodology (equal); project administration (equal); supervision (lead); visualisation (supporting); writing – original draft (equal); writing – review and editing (equal).

## Funding

This work was supported by the Scottish Government, National Institutes of Health (1R01DA059822‐01A1) and National Institute for Health and Care Research.

## Ethics Statement

The authors have nothing to report.

## Conflicts of Interest

The authors declare no conflicts of interest.

## Data Availability

The data that support the findings of this study are available on request from the corresponding author. The data are not publicly available due to privacy or ethical restrictions.

## Appendix A Definition of Events Used in the Model

Two event types were included:

**Fatal drug‐related poisonings:** we included accidental fatal drug‐related poisonings where:

- ICD‐10 code F11.2, F11.9 or X42 was reported as the main underlying cause of death, AND
- toxicology reports indicated that heroin/morphine, methadone or buprenorphine were implicated in or potentially contributed to the death, AND
- the death did not occur among people who had been prescribed strong opioid analgesics on a long‐term basis

We purposefully excluded suicides, deaths where opioids were not explicitly recorded on toxicology reports, and deaths among people prescribed strong opioid analgesics on a long‐term basis.

**Non‐fatal drug‐related poisonings:** we included non‐fatal drug‐related poisonings that

- led to acute hospital admissions, and
- ICD‐10 code indicating poisoning by opium, heroin or methadone (codes T40.0, T40.1, T40.3 in main or secondary position of the first episode), AND
- the hospital admissions did not occur among people who had been prescribed strong opioid analgesics on a long‐term basis

We excluded records for which the ICD‐10 code did not explicitly indicate opioid involvement, even if the presenting symptoms were consistent with opioid‐related overdose. We also excluded admissions that resulted in death, as these events were counted within the mortality data, to avoid double counting.

## Appendix B Supplementary Tables and Figures

**TABLE B1:** Estimated prevalence of opioid dependence among the population aged 15–69 years in Scotland; overall and by sex; 2014/15 to 2023/24. Number in brackets are 95% credible intervals (CrI). Implied % observed: Percentage of the estimated population with opioid dependence who are in the baseline cohort; Implied % opioid agonist therapy (OAT) exposure: Percentage of the estimated population with opioid dependence who received at least one opioid agonist therapy prescription during the year.

Overall	Estimated number of people with opioid dependence (95% CrI)	Prevalence (95% CrI)	Implied %, Observed (95% CrI)	Implied %, OAT Exposure (95% CrI)
2014/15	47,700 (45,800‐49,800)	1.25% (1.19%–1.30%)	76% (73%–79%)	61% (59%–64%)
2015/16	46,700 (45,000‐48,600)	1.21% (1.17%–1.26%)	78% (75%–81%)	63% (61%–66%)
2016/17	45,700 (44,200‐47,300)	1.18% (1.14%–1.22%)	80% (77%–83%)	66% (63%–68%)
2017/18	44,400 (43,200‐45,800)	1.15% (1.12%–1.19%)	82% (79%–84%)	68% (66%–70%)
2018/19	44,400 (43,300‐45,700)	1.16% (1.12%–1.19%)	81% (79%–83%)	67% (65%–69%)
2019/20	46,300 (44,800‐47,900)	1.20% (1.16%–1.24%)	77% (75%–80%)	64% (62%–66%)
2020/21	46,200 (44,800‐47,600)	1.20% (1.16%–1.24%)	78% (75%–80%)	65% (63%–67%)
2021/22	43,200 (42,000‐44,600)	1.12% (1.09%–1.16%)	82% (79%–84%)	69% (67%–71%)
2022/23	42,100 (40,800‐43,600)	1.10% (1.06%–1.14%)	83% (80%–86%)	70% (67%–72%)
2023/24	43,600 (42,000‐45,400)	1.13% (1.09%–1.18%)	78% (75%–81%)	65% (62%–67%)

Females	Number (95% CrI)	Prevalence (95% CrI)	Implied %, Observed (95% CrI)	Implied %, OAT Exposure (95% CrI)
2014/15	15,100 (14,500‐15,700)	0.77% (0.74%–0.81%)	80% (77%–83%)	64% (62%–67%)
2015/16	14,800 (14,300‐15,400)	0.75% (0.73%–0.78%)	81% (78%–84%)	66% (63%–68%)
2016/17	14,400 (14,000‐15,000)	0.73% (0.71%–0.76%)	83% (80%–86%)	68% (65%–70%)
2017/18	14,000 (13,600‐14,500)	0.72% (0.69%–0.74%)	85% (82%–88%)	70% (68%–72%)
2018/19	13,900 (13,500‐14,400)	0.71% (0.69%–0.73%)	84% (81%–87%)	70% (67%–72%)
2019/20	14,400 (14,000‐14,900)	0.74% (0.71%–0.76%)	81% (78%–83%)	67% (65%–69%)
2020/21	14,300 (13,900‐14,800)	0.73% (0.71%–0.76%)	81% (78%–84%)	68% (66%–70%)
2021/22	13,500 (13,100‐14,000)	0.69% (0.67%–0.71%)	85% (82%–87%)	72% (69%–74%)
2022/23	13,200 (12,800‐13,600)	0.67% (0.65%–0.70%)	85% (83%–88%)	72% (70%–74%)
2023/24	13,500 (13,000‐14,100)	0.68% (0.66%–0.71%)	82% (78%–85%)	68% (65%–70%)

Males	Number (95% CrI)	Prevalence (95% CrI)	Implied %, Observed (95% CrI)	Implied %, OAT Exposure (95% CrI)
2014/15	32,600 (31,200‐34,200)	1.73% (1.66%–1.82%)	74% (71%–77%)	60% (57%–63%)
2015/16	31,900 (30,700‐33,300)	1.69% (1.62%–1.77%)	76% (73%–79%)	62% (60%–65%)
2016/17	31,200 (30,100‐32,500)	1.65% (1.59%–1.71%)	78% (75%–81%)	65% (62%–67%)
2017/18	30,400 (29,500‐31,400)	1.61% (1.56%–1.66%)	81% (78%–83%)	67% (65%–69%)
2018/19	30,500 (29,600‐31,500)	1.61% (1.57%–1.66%)	80% (77%–82%)	66% (64%–68%)
2019/20	31,900 (30,800‐33,100)	1.68% (1.63%–1.75%)	76% (73%–79%)	63% (61%–65%)
2020/21	31,800 (30,800‐32,900)	1.68% (1.63%–1.74%)	76% (74%–79%)	63% (61%–65%)
2021/22	29,700 (28,800‐30,800)	1.57% (1.52%–1.62%)	80% (78%–83%)	68% (65%–70%)
2022/23	28,900 (28,000‐30,000)	1.54% (1.49%–1.60%)	82% (79%–84%)	69% (66%–71%)
2023/24	30,100 (28,900‐31,500)	1.59% (1.53%–1.66%)	77% (74%–80%)	64% (61%–66%)

**TABLE B2:** Estimated prevalence (%) of opioid dependence by age‐sex group; Data Sources: Opioid‐related deaths, Opioid‐related hospitalisations, joint (both data sources); 2014/15 to 2023/24.

FY	Sex	Age	Deaths	Joint	Hospital admissions
2014/15	Females	15–34	0.95% (0.89%, 1.02%)	0.92% (0.88%, 0.97%)	0.91% (0.85%, 0.98%)
2014/15	Males	15–34	1.74% (1.56%, 1.95%)	1.55% (1.44%, 1.66%)	1.40% (1.29%, 1.55%)
2015/16	Females	15–34	0.84% (0.78%, 0.90%)	0.82% (0.79%, 0.87%)	0.83% (0.78%, 0.90%)
2015/16	Males	15–34	1.52% (1.37%, 1.71%)	1.39% (1.29%, 1.49%)	1.29% (1.19%, 1.42%)
2016/17	Females	15–34	0.74% (0.69%, 0.79%)	0.73% (0.69%, 0.76%)	0.73% (0.68%, 0.79%)
2016/17	Males	15–34	1.34% (1.21%, 1.49%)	1.21% (1.13%, 1.30%)	1.12% (1.02%, 1.24%)
2017/18	Females	15–34	0.68% (0.64%, 0.73%)	0.65% (0.62%, 0.69%)	0.64% (0.60%, 0.68%)
2017/18	Males	15–34	1.25% (1.13%, 1.04%)	1.10% (1.03%, 1.17%)	0.97% (0.89%, 1.06%)
2018/19	Females	15–34	0.61% (0.58%, 0.65%)	0.60% (0.57%, 0.63%)	0.60% (0.56%, 0.65%)
2018/19	Males	15–34	1.17% (1.06%, 1.29%)	1.04% (0.97%, 1.11%)	0.95% (0.87%, 1.05%)
2019/20	Females	15–34	0.61% (0.57%, 0.67%)	0.60% (0.56%, 0.63%)	0.59% (0.54%, 0.65%)
2019/20	Males	15–34	1.27% (1.14%, 1.43%)	1.11% (1.03%, 1.21%)	0.99% (0.89%, 1.11%)
2020/21	Females	15–34	0.59% (0.54%, 0.63%)	0.56% (0.53%, 0.60%)	0.56% (0.51%, 0.62%)
2020/21	Males	15–34	1.25% (1.13%, 1.40%)	1.08% (1.00%, 1.16%)	0.96% (0.86%, 1.08%)
2021/22	Females	15–34	0.50% (0.46%, 0.54%)	0.48% (0.45%, 0.51%)	0.46% (0.42%, 0.51%)
2021/22	Males	15–34	1.05% (0.94%, 1.18%)	0.92% (0.85%, 0.99%)	0.80% (0.72%, 0.90%)
2022/23	Females	15–34	0.45% (0.41%, 0.49%)	0.43% (0.40%, 0.46%)	0.42% (0.38%, 0.47%)
2022/23	Males	15–34	0.98% (0.87%, 1.11%)	0.86% (0.79%, 0.94%)	0.76% (0.68%, 0.87%)
2023/24	Females	15–34	0.45% (0.41%, 0.50%)	0.43% (0.40%, 0.47%)	0.41% (0.36%, 0.48%)
2023/24	Males	15–34	1.08% (0.95%, 1.24%)	0.92% (0.84%, 1.02%)	0.78% (0.67%, 0.93%)

FY	Sex	Age	Deaths	Joint	Hospital admissions
2014/15	Females	35–49	1.30% (1.24%, 1.36%)	1.34% (1.29%, 1.40%)	1.45% (1.35%, 1.59%)
2014/15	Males	35–49	3.50% (3.35%, 3.68%)	3.54% (3.42%, 3.68%)	3.64% (3.42%, 3.92%)
2015/16	Females	35–49	1.36% (1.31%, 1.41%)	1.40% (1.35%, 1.46%)	1.49% (1.40%, 1.61%)
2015/16	Males	35–49	3.55% (3.41%, 3.70%)	3.59% (3.48%, 3.72%)	3.66% (3.47%, 3.90%)
2016/17	Females	35–49	1.40% (1.36%, 1.45%)	1.45% (1.40%, 1.50%)	1.58% (1.48%, 1.71%)
2016/17	Males	35–49	3.57% (3.46%, 3.70%)	3.63% (3.53%, 3.75%)	3.81% (3.60%, 4.09%)
2017/18	Females	35–49	1.45% (1.40%, 1.50%)	1.48% (1.44%, 1.52%)	1.55% (1.47%, 1.64%)
2017/18	Males	35–49	3.62% (3.52%, 3.74%)	3.64% (3.55%, 3.74%)	3.68% (3.53%, 3.87%)
2018/19	Females	35–49	1.47% (1.43%, 1.51%)	1.50% (1.46%, 1.55%)	1.57% (1.49%, 1.66%)
2018/19	Males	35–49	3.58% (3.48%, 3.68%)	3.60% (3.51%, 3.69%)	3.65% (3.49%, 3.82%)
2019/20	Females	35–49	1.51% (1.47%, 1.56%)	1.56% (1.52%, 1.61%)	1.70% (1.60%, 1.82%)
2019/20	Males	35–49	3.61% (3.49%, 3.73%)	3.67% (3.56%, 3.78%)	3.83% (3.62%, 4.09%)
2020/21	Females	35–49	1.52% (1.48%, 1.57%)	1.56% (1.52%, 1.61%)	1.66% (1.57%, 1.77%)
2020/21	Males	35–49	3.54% (3.43%, 3.65%)	3.57% (3.47%, 3.67%)	3.66% (3.47%, 3.88%)
2021/22	Females	35–49	1.48% (1.44%, 1.53%)	1.51% (1.46%, 1.55%)	1.55% (1.47%, 1.65%)
2021/22	Males	35–49	3.28% (3.18%, 3.40%)	3.29% (3.20%, 3.39%)	3.29% (3.14%, 3.49%)
2022/23	Females	35–49	1.45% (1.41%, 1.49%)	1.47% (1.43%, 1.52%)	1.54% (1.46%, 1.65%)
2022/23	Males	35–49	3.17% (3.07%, 3.28%)	3.18% (3.09%, 3.28%)	3.25% (3.07%, 3.48%)
2023/24	Females	35–49	1.43% (1.38%, 1.48%)	1.46% (1.41%, 1.51%)	1.54% (1.43%, 1.67%)
2023/24	Males	35–49	3.11% (3.00%, 3.24%)	3.13% (3.03%, 3.25%)	3.18% (2.97%, 3.48%)

FY	Sex	Age	Deaths	Joint	Hospital admissions
2014/15	Females	50–69	0.17% (0.15%, 0.19%)	0.18% (0.16%, 0.21%)	0.22% (0.18%, 0.28%)
2014/15	Males	50–69	0.49% (0.43%, 0.56%)	0.51% (0.45%, 0.57%)	0.55% (0.46%, 0.68%)
2015/16	Females	50–69	0.18% (0.17%, 0.21%)	0.20% (0.18%, 0.22%)	0.23% (0.19%, 0.28%)
2015/16	Males	50–69	0.53% (0.48%, 0.61%)	0.54% (0.50%, 0.60%)	0.58% (0.50%, 0.68%)
2016/17	Females	50–69	0.19% (0.18%, 0.21%)	0.21% (0.19%, 0.23%)	0.25% (0.21%, 0.31%)
2016/17	Males	50–69	0.56% (0.52%, 0.62%)	0.59% (0.55%, 0.64%)	0.65% (0.57%, 0.78%)
2017/18	Females	50–69	0.21% (0.19%, 0.22%)	0.21% (0.20%, 0.23%)	0.23% (0.20%, 0.26%)
2017/18	Males	50–69	0.62% (0.57%, 0.66%)	0.61% (0.58%, 0.65%)	0.63% (0.57%, 0.70%)
2018/19	Females	50–69	0.24% (0.22%, 0.26%)	0.25% (0.23%, 0.27%)	0.27% (0.24%, 0.31%)
2018/19	Males	50–69	0.73% (0.68%, 0.78%)	0.73% (0.69%, 0.77%)	0.74% (0.68%, 0.82%)
2019/20	Females	50–69	0.27% (0.25%, 0.28%)	0.28% (0.26%, 0.29%)	0.31% (0.27%, 0.35%)
2019/20	Males	50–69	0.80% (0.76%, 0.86%)	0.81% (0.77%, 0.86%)	0.85% (0.77%, 0.95%)
2020/21	Females	50–69	0.29% (0.27%, 0.31%)	0.30% (0.28%, 0.32%)	0.34% (0.31%, 0.40%)
2020/21	Males	50–69	0.90% (0.86%, 0.95%)	0.92% (0.88%, 0.96%)	0.97% (0.89%, 1.09%)
2021/22	Females	50–69	0.31% (0.29%, 0.33%)	0.31% (0.29%, 0.33%)	0.32% (0.29%, 0.35%)
2021/22	Males	50–69	0.98% (0.93%, 1.03%)	0.96% (0.92%, 1.01%)	0.95% (0.89%, 1.02%)
2022/23	Females	50–69	0.34% (0.32%, 0.36%)	0.34% (0.32%, 0.37%)	0.34% (0.31%, 0.38%)
2022/23	Males	50–69	1.07% (1.01%, 1.14%)	1.05% (1.00%, 1.12%)	1.01% (0.95%, 1.10%)
2023/24	Females	50–69	0.38% (0.35%, 0.40%)	0.38% (0.36%, 0.41%)	0.38% (0.34%, 0.42%)
2023/24	Males	50–69	1.18% (1.11%, 1.26%)	1.16% (1.10%, 1.23%)	1.10% (1.02%, 1.20%)

Abbreviation: FY, financial year.
